# Key community eye health messages

**Published:** 2017-08-07

## A comprehensive school eye health programme:

**Figure:**
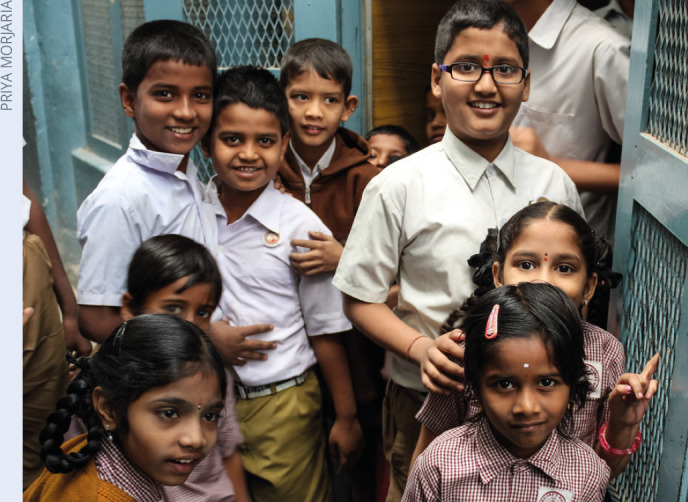

- Should be integrated into a broader school health programme
- Requires a goal that will result in positive change
- Must have the engagement of the ministries of health and education
- Needs ‘SMART’ objectives for each component of the programme

## A key issue in a school eye health programme is that children may not wear their spectacles.

**Figure:**
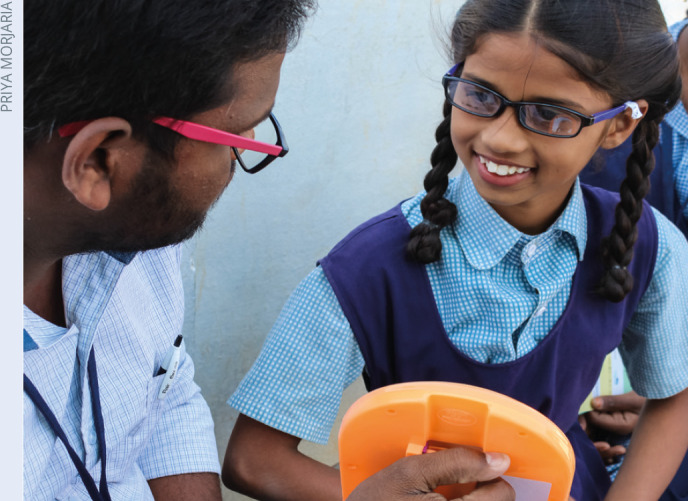

- Parents should understand why a child needs spectacles
- The child's vision must improve with correction
- The child must feel comfortable wearing spectacles and like the frames
- The spectacles should be affordable
- Teachers should encourage children to wear their glasses

## A school eye health programme requires careful planning, with a goal and specific objectives which address:

**Figure:**
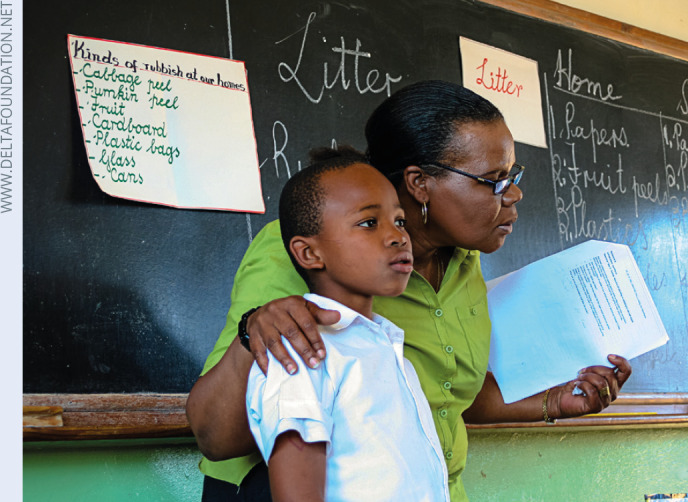

- School children with refractive errors and other eye conditions
- Teachers who may themselves have refractive errors or other eye conditions
- Broader eye health education: children can act as ‘agents of change’ in their families and communities

